# Residential distance to major roadways and cardiac structure in African Americans: cross-sectional results from the Jackson Heart Study

**DOI:** 10.1186/s12940-017-0226-4

**Published:** 2017-03-08

**Authors:** Anne M. Weaver, Gregory A. Wellenius, Wen-Chih Wu, DeMarc A. Hickson, Masoor Kamalesh, Yi Wang

**Affiliations:** 1 Richard M. Fairbanks School of Public Health, Indiana University, 1050 Wishard Blvd., RG 6082, Indianapolis, IN 46202 USA; 2 The School of Public Health at Brown University, 121 South Main Street, Providence, RI 02903 USA; 3 Jackson State University School of Public Health Initiative, 350 West Woodrow Wilson Drive, Jackson Medical Mall, Suite 320, Jackson, MS 39213 USA; 4 Department of Cardiology, Richard L. Roudebush VA Medical Center, 1481 W 10th St., Indianapolis, IN 46202 USA

**Keywords:** Air pollution, Cardiac structure, Cardiovascular disease, African Americans, Heart failure

## Abstract

**Background:**

Heart failure (HF) is a significant source of morbidity and mortality among African Americans. Ambient air pollution, including from traffic, is associated with HF, but the mechanisms remain unknown. The objectives of this study were to estimate the cross-sectional associations between residential distance to major roadways with markers of cardiac structure: left ventricular (LV) mass index, LV end-diastolic diameter, LV end-systolic diameter, and LV hypertrophy among African Americans.

**Methods:**

We studied baseline participants of the Jackson Heart Study (recruited 2000–2004), a prospective cohort of cardiovascular disease (CVD) among African Americans living in Jackson, Mississippi, USA. All cardiac measures were assessed from echocardiograms. We assessed the associations between residential distance to roads and cardiac structure indicators using multivariable linear regression or multivariable logistic regression, adjusting for potential confounders.

**Results:**

Among 4826 participants, residential distance to road was <150 m for 103 participants, 150–299 m for 158, 300–999 for 1156, and ≥1000 m for 3409. Those who lived <150 m from a major road had mean 1.2 mm (95% CI 0.2, 2.1) greater LV diameter at end-systole compared to those who lived ≥1000 m. We did not observe statistically significant associations between distance to roads and LV mass index, LV end-diastolic diameter, or LV hypertrophy. Results did not materially change after additional adjustment for hypertension and diabetes or exclusion of those with CVD at baseline; results strengthened when modeling distance to A1 roads (such as interstate highways) as the exposure of interest.

**Conclusions:**

We found that residential distance to roads may be associated with LV end-systolic diameter, a marker of systolic dysfunction, in this cohort of African Americans, suggesting a potential mechanism by which exposure to traffic pollution increases the risk of HF.

**Electronic supplementary material:**

The online version of this article (doi:10.1186/s12940-017-0226-4) contains supplementary material, which is available to authorized users.

## Background

Heart failure (HF) is a common cause of morbidity and mortality in the US, listed on one of every nine death certificates in 2009 [1]. Although mortality from all forms of cardiovascular disease (CVD) combined has decreased by nearly one third from 1999 to 2009, the number of deaths from HF in 2009 was approximately the same as it was in 1995 [1]. Exposure to short-term and long-term ambient air pollution has been shown to increase risk of cardiovascular morbidity and mortality, including from HF [2]. A reduction of 3.9 μg/m^3^ in short-term exposure to fine particulate matter (PM_2.5_–particulates < 2.5 μm in diameter) air pollution across the US is estimated to prevent approximately 8000 HF hospitalizations and a $300 million reduction in healthcare costs every year [3]. Long-term exposure to common traffic-related air pollutants such as PM_10_ (particles <10 μm in diameter, including coarse and fine particulate matter), PM_2.5_, nitrogen dioxide (NO_2_), and black smoke has been shown to be associated with increased risk [4, 5] and mortality from heart failure [4].

Air pollution from traffic is a major contributor to ambient air pollution and to cardiovascular morbidity and mortality in urban areas [6]. Residential proximity to major roadways is a surrogate measure of long-term exposure to traffic-related air pollution and has been associated with higher cardiovascular morbidity [6], including higher risk of hypertension [7, 8], stroke [9], and 10-year all-cause mortality after myocardial infarction [10], as well as lower renal function [11]. Changes in cardiac structure, termed cardiac remodeling, in response to left ventricle (LV) diastolic and systolic dysfunction plays a central role in HF progression [12]. It is possible that exposure to air pollution can lead to chronic inflammation and/or oxidative stress, and the heart may compensate to these stressors via cardiac remodeling [3, 13–15]. Additionally, long-term exposure to air pollution may be associated with hypertension [16, 17], and elevated blood pressure has been shown to be associated with LV mass index (LVMI) [18]. Few studies have examined how long-term exposure to traffic pollution or residential proximity to roadways is associated with cardiac structure. One study in mice found increased heart size, LV end-systolic and end-diastolic diameters after long-term exposure to PM_2.5_ [15]. A study in humans enrolled in the Multi-Ethnic Study of Atherosclerosis (MESA) in the USA found that those who resided <50 m from a major roadway had a 1.4 g/m^2^ greater LVMI compared to those who resided >150 m from a major roadway [13]. However, the SALIA study in Germany found no association between NO_2_ or PM_10_ with LVMI [19].

African Americans are at an increased risk of HF incidence and mortality compared to white Americans [1, 20–22]. African Americans also have higher prevalence of cardiovascular risk factors, such as diabetes mellitus and hypertension [1]. No study to date has investigated the potential association between residential proximity to major roadways and cardiac remodeling specifically in African Americans. The state of Mississippi has the highest proportion of African Americans and the highest rate of death from cardiovascular disease of any state in the US [1, 23]. In this study, we evaluated the cross-sectional association between residential proximity to major roadways and indicators of cardiac structure among African Americans residing in Mississippi who were baseline participants of the Jackson Heart Study (JHS).

## Methods

Between 2000 and 2004, JHS recruited a total of 5301 non-institutionalized African American men and women aged 21 years and older, residing in the tri-county Jackson, Mississippi, USA Metropolitan Statistical Area (MSA), as previously described [24, 25]. The JHS cohort has been shown to be representative of the underlying African American population living in the Jackson MSA [26]. Upon enrollment, participants completed an in-home interview followed by a clinic visit [24, 25].

### Residential proximity to major roadways

We calculated residential distance to the nearest major roadway as a marker of long-term exposure to traffic-related pollution for each participant. We used ArcGIS (version 9.2; ESRI Inc., Redlands, CA) to geocode participants’ addresses and calculate the straight-line distance from each residence to the nearest major roadway, defined as roads with U.S. Census Feature Class Code A1 (primary highway with limited access—only accessible via ramps) or A2 (primary road without limited access), as previously described [27–29]. Topologically Integrated Geographic Encoding and Referencing (TIGER) and 2000 US Census data were used for geocoding roads and addresses [30].

### Indicators of cardiac structure

In this study, we considered the following indicators of cardiac structure as outcomes: left ventricular mass index (LVMI), LV hypertrophy, LV end-diastolic diameter, and LV end-systolic diameter. LVMI is a continuous measure of cardiac remodeling, LV hypertrophy is a cutpoint at which LVMI is pathologic, LV end-diastolic and end-systolic diameter are indicators of systolic function. These indicators are all commonly measured during electrocardiograms and are used as preclinical markers of heart disease. Long-term exposure to traffic pollution, over years, may lead to noticeable changes in these indicators. During the baseline clinic visit, JHS participants underwent prioritized 30-min two-dimensional and M-mode echocardiography with parasternal, apical, and subcostal windows [25]. Echocardiography was performed using a Sonos 4500 echocardiograph (Philips Medical Systems) by a certified, experienced cardiac ultrasonography technician [25, 31]. All echocardiograms were analyzed using the American Society of Echocardiography conventions [31, 32]; we used measurements from two-dimensional echocardiograms to define all outcomes. Left ventricular mass was determined as previously described [33–35]. Left ventricular mass index was defined as LV mass indexed to height^2.7^ to account for body size [33–35]. LV hypertrophy was defined as an LVMI value ≥51 g/m^2.7^, a threshold previously validated in the Jackson subcohort of the Atherosclerosis Risk in the Communities (ARIC) study [35].

### Covariates

At baseline, participants were given an in-person interview, detailing demographics, health history, lifestyle factors, and healthcare access. Shortly thereafter, participants were given a clinical exam. Details of the baseline interview and exam are described elsewhere [25]. During the clinical exam, medical history was assessed by interview, anthropometrics were measured, sitting blood pressure was measured, and blood samples were drawn and analyzed.

We examined the variables described below as potential covariates. Age and sex were based on self-report. Body mass index (BMI) was calculated from measured weight (kilograms) divided by measured height (meters) squared. Education was categorized as the highest level of education completed. Type of medical insurance was categorized as none, private only, public only, and both public and private. Occupation was categorized based on Sims et al [36] and the distribution in our sample. Household income level (low, lower-middle, upper-middle, and high) was determined based on self-reported income, family size, number of children <18 years old, and the US Census designated poverty level for the year of data collection [37]. Neighborhood socioeconomic status (NSES) was determined at the census tract level based on six variables derived from the US Census (www.census.gov): percent of adults >25 years with less than high school education, percent of males who were unemployed, percent of households with income below the poverty line, percent of households receiving public assistance, percent of households with children headed by a woman, and median household income, as described in Dubowitz et al [38]. NSES was then converted to a z score, as described by Diez Roux et al [39]. Smoking status and alcohol consumption in the past 12 months were based on self-report. Nutritional status and physical activity were categorized as poor, intermediate, or ideal according to Life’s Simple 7 criteria [40, 41]. Participants were classified as having hyperlipidemia if total cholesterol was ≥ 240 mg/dl or low-density lipoprotein cholesterol level was ≥ 160 mg/dl or they were taking lipid-lowering medications [1]. Participants were classified as hypertensive if their supine blood pressure at the baseline clinical examination was ≥140/90 mmHg or they were taking blood pressure lowering medication [42, 43]. Participants were classified as having diabetes if they had measured hemoglobin A1c levels ≥ 6.5%, had fasting glucose measurement ≥ 126 mg/dl, or were taking any diabetes medications [44]. Medication assessments were based both on self-report and inventory of medications taken in the 2 weeks prior to the clinic visit.

### Statistical analyses

Our analytic sample excluded participants who were missing all LV measurements from echocardiograms, as well as those with aortic or mitral stenosis or regurgitation, as these conditions may affect LV measurements [45]. We included only those participants with valid geocoded address data at the street level.

**Fig. 1:**
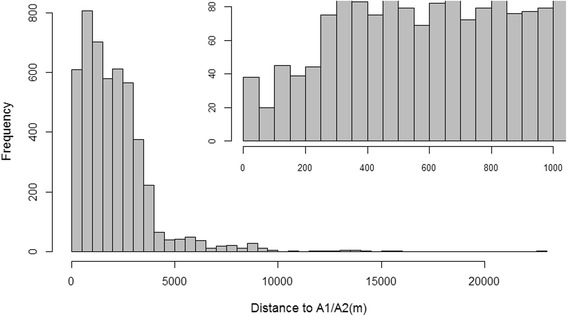
Distribution of residential distance to A1 or A2 roads in the Jackson Heart Study in cutpoints of 500 m (*N* = 4826). Inset: distribution of residential distance to A1 or A2 roads among those who lived within 1000 m of A1 or A2 roads in cutpoints of 50 m (*n* = 1417)

We examined residential distance to A1 or A2 roads in categories of <150 m, 150–299 m, 300–999 m, and ≥1000 m and as a continuous variable with natural log transformation. We chose cutpoints of 150 m and 300 m because ultrafine particulate matter concentration from highway traffic pollution has been shown to decrease by 50% at 150 m from roads and fade into background levels after 300 m [46]. We also chose to include a cutpoint of 1000 m, as those who live more than 1 km from a major road may be different from those who live nearer, for instance, they may live in more rural areas. We chose to log-transform distance to roads due to the right skew of the distribution (Fig. 1), reflecting non-linear distance decay of air pollutants.

We examined the associations between residential distance to A1 or A2 roads and continuous indicators of cardiac structure (all outcomes except LV hypertrophy) using multivariable linear regression adjusting for confounders. We examined the associations between residential distance to A1 or A2 roads and LV hypertrophy using multivariable logistic regression adjusting for confounders. We conducted a test for trend for categorical distance to roads.

We decided *a priori* to adjust all models for potential confounding by age, sex, and BMI. We determined additional covariates by conducting bivariate analyses (chi-square tests or ANOVA, as appropriate) between the potential covariates and categories of residential proximity to A1 or A2 roads (<150 m, 150–299 m, 300–999 m, ≥1000 m) and indicators of cardiac structure. We adjusted models for those covariates that were associated with residential proximity to A1 or A2 roads and cardiac structure at a *p*-value of 0.05 and were not collinear with other covariates (variance inflation factor <10). In the case of collinearity, we included the variable with the least missing data. We adjusted models incrementally, first for anthropomentric and health factors (age, sex, BMI, alcohol consumption, type of medical insurance, and smoking status), then we added socioeconomic factors (education, occupation, neighborhood SES) for the fully adjusted models. Although associated with exposure at the *p* = 0.05 level, we did not adjust for household income status due to a great number of participants (*n* = 462) with missing values. Post-hoc analyses showed additional adjustment for household income did not substantially change estimates of association. Comorbid conditions, such as hypertension and diabetes, may be confounders or mediators in the relationship between residential distance to major roadway and heart failure. We calculated models with and without additional adjustment for diabetes and hypertension. We additionally considered the possibility that diabetes and hypertension may interact with residential distance to roads to influence cardiac structure; we tested interaction terms for diabetes and hypertension in fully adjusted models.

### Sensitivity analysis

We conducted additional sensitivity analyses. First, since many (*n* = 254) participants were missing all LV measurements from echocardiograms, we compared descriptive characteristics of those who were and were not missing these data. Second, we redefined the exposure (residential distance to major roadway) as residential distance to A1 (primary highway with limited access) roads as A1 roads are typically busier and have more heavy-duty diesel traffic compared to A2 roads. We classified residential distance to A1 roads as <150 m, 150–299 m, 300–999 m, and ≥1000 m, as well as log-transformed distance as a continuous variable, similar to primary analyses, adjusting for the same covariates as in primary analyses. Third, we considered alternate classifications of residential distance to A1 or A2 roads, including cutpoints of 100 m, 200 m, and 400 m, modeling distance to roads as an un-transformed continuous variable, modeling the inverse of distance to roads, and truncating distance to roads at 400 m, as distances beyond 400 m are unlikely to be affected by traffic-related air pollution. Fourth, we excluded participants with CVD at baseline, defined as a history of myocardial infarction (based on self-report or electrocardiogram), carotid angioplasty (self-report), or stroke (based on self-report of doctor diagnosis or experiencing any of following symptoms with sudden onset and lasting at least 24 h: loss of speech, loss of vision, double vision, numbness or tingling, paralysis or weakness, or dizziness or loss of balance). Fifth, we limited our analyses to those participants who resided in urban areas, defined by the 2010 US Census Urban and Rural Classification (www.census.gov). Sixth, we restricted our analyses to those living within 5000 m of roads. All analyses were conducted using SAS version 9.4; results were considered statistically significant at a two-sided *p*-value of <0.05. We also plotted natural splines with 3° of freedom for the associations between log-transformed distance to A1 or A2 roads and LV end-systolic diameter, LV end-diastolic diameter, and LVMI, adjusting for covariates in the final regression model using R version 3.3.1 (The R Foundation for Statistical Computing, Vienna, Austria).

## Results

The Jackson Heart Study recruited a total of 5301 participants at baseline. We excluded 254 (5%) participants who were missing all LV echocardiographic measurements, 211 (4%) participants whose baseline addresses could not be geocoded, 5 (0.09%) participants with severe aortic stenosis, 5 participants with severe aortic regurgitation, and 4 (0.08%) participants who were missing information on residential distance to A1 and A2 roads, making our final sample size 4826 participants. The distribution of residential distance to A1 and A2 roads is shown in Fig. 1. The majority (70.6%) of participants lived >1000 m from a major roadway, with only 2.1% living <150 m from a major roadway (Table 1). Those residing nearest to A1 or A2 roads (<150 m) tended to have less education (34.3% had less than high school education), lower income (21.1% were low-income), and more likely to have public health insurance. Other participant characteristics such as age, sex and BMI also varied according to roadway proximity, but not necessarily in a monotonic manner. Overall, less than half of participants consumed alcohol in the past 12 months; consumption was lowest among those living <150 m from A1 or A2 roads (39.0%). Those who lived <150 m from A1 or A2 roads had the lowest prevalence of hypertension (55.9%). Among those residing <150 m, 150–299 m, 300–99 m, and ≥1000 m from A1 or A2 roads, LVMI was 38.9, 35.8, 35.7, and 36.4 g/m^2.7^, respectively. In bivariate analyses, there who lived nearest roads had the highest prevalence of LV hypertrophy (15.5%); there were no statistically significant differences in LV end-diastolic diameter, or LV end-systolic diameter by residential distance to A1 or A2 roads.

**Table 1:** Descriptive characteristics of JHS participants by distance to A1 or A2 road (*N* = 4826)

	<150 m (*n* = 103)	150–299 m (*n* = 158)	300–999 m (*n* = 1156)	≥1000 m (*n* = 3409)
Characteristic	Mean (SD) or %	Mean (SD) or %	Mean (SD) or %	Mean (SD) or %
Age, years, mean (SD)*	55.9 (13.1)	58.8 (12.1)	54.6 (12.1)	55.5 (13.0)
Female	68.0	65.2	62.0	64.1
BMI (kg/m^2^), mean (SD)	33.1 (8.4)	30.9 (6.7)	31.6 (7.0)	31.7 (7.2)
Highest level of education completed*				
Less than high school	34.3	20.9	15.9	20.5
High school/GED	30.4	33.5	34.2	38.4
College degree/certificate	24.5	22.2	29.6	26.4
Graduate/professional school	10.8	23.4	20.3	14.7
Household income status*				
Low	21.1	6.9	12.3	16.4
Lower-middle	26.3	26.0	19.5	25.8
Upper-middle	18.4	30.0	30.7	30.2
High	34.2	37.4	37.5	27.7
Neighborhood SES z-score, mean (SD)^a^*	0.6 (6.4)	1.6 (6.6)	0.8 (5.1)	−0.8 (4.7)
Medical Insurance*				
None	13.6	12.1	13.0	13.6
Private	41.8	50.3	55.5	49.6
Public	27.2	20.4	17.2	22.6
Public and private	16.5	17.2	14.4	14.2
Smoking status				
Never	64.1	74.7	69.3	67.5
Former	15.5	15.8	19.1	19.3
Current	20.4	9.5	11.6	13.2
Physical activity^b^				
Poor	49.5	48.7	46.3	50.4
Intermediate	27.2	31.1	34.2	30.8
Ideal	23.3	20.3	19.5	18.8
Alcohol consumption, past 12 months*	39.0	38.5	47.3	43.7
Occupation*				
Management/professional	27.2	42.4	42.2	33.7
Service	25.2	23.4	22.9	25.7
Sales	18.5	15.2	16.6	17.7
Other	29.1	19.0	18.4	22.9
Hypertension*	55.9	67.7	57.6	62.3
Diabetes	16.5	22.4	21.6	22.1
Hyperlipidemia	32.4	29.5	29.4	29.3
History of CVD, %	11.7	12.7	8.6	11.0
LVMI, g/m^2.7^, mean (SD)*	38.9 (14.0)	35.8 (9.8)	35.7 (9.4)	36.4 (12.5)
LV hypertrophy, %*	15.5	6.3	6.2	8.6
LV end-diastolic diameter, mm, mean (SD)	49.2 (4.5)	48.2 (4.3)	48.6 (4.5)	48.4 (4.6)
LV end-systolic diameter, mm, mean (SD)	30.9 (4.9)	29.5 (4.6)	29.8 (4.8)	29.8 (5.0)

Percent missing: BMI (0.1), education level (0.3), household income (15.3), medical insurance (0.4), smoking status (0.7), physical activity (0.06), alcohol consumption (2.6), occupation (0.06), hypertension (0.06), diabetes (1.1), hyperlipidemia (1.4), LVMI (0.1), LV hypertrophy (0.1), LV systolic diameter (0.1)

^a^ Neighborhood socioeconomic status determined using methods described by Dubowitz [50]

^b^Physical activity as described in the American Heart Association’s Life’s Simple 7 [40, 41]

* *p* < 0.05 (chi-square or ANOVA)

In unadjusted models, we observed the following associations: those who lived <150 m from A1 or A2 roads had 2.5 g/m^2.7^ greater LVMI (95% CI 0.1, 4.8), a nearly two-fold greater odds of LV hypertrophy (OR 1.97, 95% CI 1.14, 3.40), and a 1.1 mm larger LV end-systolic diameter (95% CI 0.1, 2.1) (results not shown). However, the associations between residential distance to roads and LVMI and LV hypertrophy were attenuated and no longer statistically significant after adjustment for covariates. After adjustment for anthropometric and health covariates, point estimates of association did not substantially change with additional adjustment for socioeconomic covariates. In the fully adjusted model, those who lived <150 m from A1 or A2 roads had a statistically significant 1.2 mm (95% CI 0.2 mm, 2.1 mm) greater LV end-systolic diameter, compared to those ≥1000 m (Table 2). We did not observe statistically significant associations between residential distance to A1 or A2 roads with LVMI, LV hypertrophy, or LV end-diastolic diameter. Results were similar with additional adjustment for hypertension and diabetes (results not shown). We did not observe any statistically significant *p*-values for the trend of categories of distance to road and cardiac structure (results not shown). We observed statistically significant *p* values for interaction for the associations between distance to A1 or A2 roads and diabetes for LVMI (beta = -0.9, 95% CI -1.7, 0.1) and LV hypertrophy (OR_Int_ = 0.78, 95% CI 0.62, 0.99) and between distance to A1 or A2 roads and hypertension for LVMI (beta = -0.7, 95% CI -1.3, -0.05). Interaction terms were negative, indicating that those with diabetes and living further from roads have smaller LVMI and lower odds of LV hypertrophy, and those who live further from roads and have hypertension have smaller LVMI.

**Table 2:** Results from linear or logistic regression of distance to roads on markers of cardiac structure in JHS (*N* = 4826)^a^

Distance to A1 or A2 road	<150 m (*n* = 103)	150–299 m (*n* = 158)	300–999 m (*n* = 1156)	≥1000 m (*n* = 3409)	Log-transformed distance to A1 or A2 road (continuous)
LVMI, g/m^2.7^, beta (95% CI)	1.3 (−0.9, 3.6)	−0.7 (−2.5, 1.1)	−0.3 (−1.1, 0.4)	REF	−0.01 (−0.3, 0.3)
LV hypertrophy, OR (95% CI)^b^	1.59 (0.88, 2.90)	0.73 (0.37, 1.42)	0.77 (0.58, 1.03)	REF	0.98 (0.88, 1.09)
LV end-diastolic diameter, mm, beta (95% CI)	0.7 (−0.2, 1.5)	−0.03 (−0.7, 0.7)	0.1 (−0.2, 0.4)	REF	−0.06 (−0.2, 0.06)
LV end-systolic diameter, mm, beta (95% CI)	1.2 (0.2, 2.1)*	−0.05 (−0.8, 0.7)	0.008 (−0.3, 0.3)	REF	−0.1 (−0.2, 0.03)
Distance to A1 road	<150 m (*n* = 34)	150–299 m (*n* = 93)	300–999 m (*n* = 793)	≥1000 m (*n* = 3906)	Log-transformed distance to A1 road (continuous)
LVMI, g/m^2.7^, beta (95% CI)	3.6 (−0.3, 7.4)	0.0 (−2.3, 2.3)	−0.06 (−0.9, 0.8)	REF	−0.2 (−0.6, 0.2)
LV hypertrophy, OR (95% CI)^b^	1.21 (0.44, 3.31)	0.82 (0.35, 1.94)	0.89 (0.64, 1.23)	REF	0.97 (0.85, 1.10)
LV end-diastolic diameter, mm, beta (95% CI)	1.7 (0.2, 3.2)*	0.09 (−0.8, 1.0)	0.03 (−0.3, 0.4)	REF	−0.08 (−0.2, 0.06)
LV end-systolic diameter, mm, beta (95% CI)	2.0 (0.4, 3.7)*	−0.08 (−1.1, 0.9)	0.04 (−0.3, 0.4)	REF	−0.08 (−0.2, 0.08)

^a^Models adjusted for age, sex, body mass index, alcohol consumption, education level, occupation, neighborhood socioeconomic status z-score, type of medical insurance, and smoking status

^b^LV hypertrophy was modeled using logistic regression; all other models use linear regression

*p < 0.05

In sensitivity analysis, those missing all LV measurements were more likely to have lower education levels (27.1% vs. 19.7% had less than high school education), lower income (21.4% vs. 15.2% were low-income), and more likely to be current smokers (19.1% vs. 12.8%) compared to those not missing all LV measurements. We did not observe any other differences in descriptive characteristics between these two groups.

In our sensitivity analyses using residential distance to A1 roads, we observed associations with LV end-diastolic diameter and LV end-systolic diameter. Those living <150 m from A1 roads had a 1.7 mm (95% CI 0.2 mm, 3.2 mm) larger LV end-diastolic diameter, and 2.0 mm (95% CI 0.4 mm, 3.7 mm) larger LV end-systolic diameter, compared to those who lived ≥1000 m from A1 roads (Table 2). We did not observe statistically significant associations between residential proximity to A1 roads and LVMI or LV hypertrophy.

We explored several methods of characterizing residential distance to A1 or A2 roads. When categorizing distance to roads as <100 m, 100–199 m, 200–399 m, or ≥ 400 m, we observed that those who lived < 100 m from A1 or A2 roads (*n* = 58) had 0.9 mm (95% CI -0.4, 2.1) larger LV end-systolic diameter and those who lived 100–199 m from A1 or A2 roads (*n* = 84) had 1.4 mm (95% CI 0.4, 2.5) larger LV end-systolic diameter; this association was only statistically significant among those who lived 100-199 m from A1 or A2 roads. Those who lived <100 m from A1 roads (*n* = 14) had 1.5 mm (−1.1, 4.1) larger LV end-systolic diameter and those who lived 100–199 m from A1 roads (*n* = 37) had 1.9 mm (95% CI 0.4–3.5) larger LV end-systolic diameter; this association was only statistically significant among those who lived 100–199 m from A1 roads (Additional file 1). We did not observe associations between continuous distance to A1 or A2 roads and indicators of cardiac structure when considering un-transformed distance, inverse distance, or truncating distance at 400 m (Additional file 2).

When excluding 505 participants with CVD at baseline, we observed similar results compared to those observed in our main analyses. Those residing <150 m from A1 or A2 roads had a borderline statistically significant 1.1 mm (95% CI 0.2 mm, 2.1 mm) larger LV end-systolic diameter, compared to those residing ≥1000 m from A1 or A2 roads (Additional file 3). We did not observe any statistically significant associations for LVMI, LV hypertrophy, or LV end-diastolic diameter when excluding those with CVD at baseline.

When limiting analyses to those who resided in urban areas (*n* = 3619), we did not observe any associations between residential distance to A1 or A2 roads and indicators of cardiac structure (results not shown). When limiting analyses to those who resided within 5000 m of A1 or A2 roads (*n* = 4578), results were similar to those in the main analysis. Those residing <150 m from A1 or A2 roads had a 1.1 mm (95% CI 0.2 mm, 2.1 mm) larger LV end-systolic diameter (*p* = 0.02) compared to those residing ≥1000 m from A1 or A2 roads (results not shown).

Spline plots showed that the associations between markers of cardiac structure and log-transformed residential distance to A1 or A2 roads appear to be approximately linear (Additional files 4, 5 and 6).

## Discussion

In this cohort of African Americans living in Mississippi, we observed some evidence of an association between residential distance to roads and cardiac remodeling; specifically, those living <150 m from A1 or A2 roads had left ventricles that were, on average, 1.2 mm larger in diameter at end-systole after adjustment for confounders. Increases in LV end-systolic diameter could lead to systolic dysfunction and, ultimately, to HF. We did not observe any statistically significant associations between residential distance to A1 or A2 roads and LVMI, LV hypertrophy, or LV end-diastolic diameter. Results were similar with further adjustment for diabetes and hypertension, indicating that diabetes and hypertension did not act as confounders in this study. However, we did observe potential interaction between diabetes and distance to roads on LVMI and LV hypertrophy and between hypertension and distance to roads on LVMI. We did not observe a dose-response relationship over categories of distance to roadway, potentially implying that one must live very near to roads (<150 m) in order for one’s cardiac structure to be adversely affected by traffic pollution. Likewise, we did not observe associations between continuous measures of distance to road and cardiac structure. Since many individuals lived far from roads, it is possible any associations were diluted when examining distance as a continuous variable.

Our results show that residential proximity to A1 or A2 roads is associated with LV end-systolic diameter and proximity to A1 roads is associated with LV end-diastolic and end-systolic diameter. When analyzing residential distance to A1 roads as the exposure of interest, associations with LVMI, LV end-diastolic, and LV end-systolic diameter were of a greater magnitude compared to analyzing residential distance to A1 and A2 roads. It is likely that A1 roads are more frequently traveled and have a higher concentration of light and heavy duty compared to A2 roads. The strengthening of our results when examining distance to A1 roads supports our conclusions of an association between distance to major roads and cardiac remodeling. We are unaware of any other epidemiologic studies that examined the association between residential proximity to roads and LV end-diastolic or end-systolic diameter in humans. However, long-term exposure to PM_2.5_ has been shown to be associated with increases in LV diastolic and systolic diameters in mice [15]. In addition, our results are consistent with the literature showing associations between air pollution exposure, cardiac remodeling, and heart failure [4, 5, 13, 47]. Proposed mechanisms of these associations include vasoconstriction and subsequent elevated blood pressure and filling pressure as responses to air pollution exposure, autonomic function, oxidative stress, or changes in vascular tone [3, 13–15]. Increased fibrosis of the myocardium, which can lead to vessel stiffening and dysfunction, has also been shown to be associated with exposure to PM_2.5_ in mice [15]. In addition to implications for air pollution, major roadways are a source of noise pollution, and noise may be associated with elevated blood pressure, which may also lead to cardiac remodeling [48].

In our study, we did not observe an association between residential proximity to A1 or A2 roads and LVMI, as Van Hee et al. did in the MESA study [13]. However, similar to our study, Ohlwein et al. failed to observe associations between LVMI and specific air pollutants [19]. Van Hee et al. categorized the nearest distance to road as <50 m from A1 or A2 roads compared to <150 m in our study. There were no participants in our study who resided <50 m from A1 or A2 roads, so we cannot directly compare our results to those of Van Hee et al. In our previous study, we did not observe associations between residential distance to road and indicators of cardiac function: left ventricular ejection fraction, E-wave velocity, isovolumic relaxation time, left atrial diameter index, or pulmonary artery systolic pressure [49]. Although we observed a clinically relevant association with LV end-systolic diameter, overall, our studies combined provide little evidence for an association between residential proximity to road and cardiac structure and function in the Jackson Heart Study.

This study has several limitations. First, we observed that those missing echocardiographic data have poorer socioeconomic indicators and are more likely to be current smokers. There is potential bias in that those who were not missing data may be in better cardiovascular health. Second,, few people (*n* = 103) resided within 150 m of A1 or A2 roads. This may have substantially reduced our power to observe associations between residential distance to A1 and A2 roads and indicators of cardiac structure. Additionally, it is possible that those living closest to roads share risk factors other than those we adjusted for, leading to residual confounding [13]. Specifically, socioeconomic indicators were different among different distances to roads; our indicators of socioeconomic status may not have fully accounted for these differences. Third, the use of residential proximity to major roadways as a surrogate of long-term exposure to traffic-related pollution may lead to exposure misclassification. This crude surrogate may not reflect spatial distribution of pollutant concentrations due to differences in traffic volume, traffic patterns, and vertical concentration gradients among addresses of an equal distance to roadways. It also did not account for other potential confounders such as traffic-related noise. However, many studies using residential proximity to major roadways had shown positive results for those living closest to major roadways consistent with associations found in studies using sophisticated air pollution modeling. Fourth, this is a cross-sectional study, from which we were not able to discern the temporal relation between long-term exposure to traffic-related pollution and atherosclerosis. Fifth, we do not have information on how long participants lived in their residences at the time of enrollment. It is possible that there could be exposure misclassification for long-term exposure to traffic-related pollution is participants moved. This is the first study to evaluate the associations between residential proximity to traffic pollution and indicators of cardiac remodeling in a large, entirely African-American population, although the findings of this study may not be generalizable to the general African-American populations in the US.

## Conclusions

Among African Americans residing in the Jackson, Mississippi metropolitan area, participants residing <150 m from A1 or A2 roads had a 1.2 mm larger LV end-systolic diameter compared to those residing ≥1000 m. This result indicates that living closer to major roadways may be associated with systolic dysfunction and cardiac remodeling in a high-risk, understudied African American population.

## Additional files

Additional file 1:
**Table A1.** Results from linear or logistic regression of distance to A1 or A2 roads, in categories of <100 m, 100–199 m, 200–399 m, ≥ 400 m on markers of cardiac structure in JHS (*N* = 4826)^a^. ^a^Models adjusted for age, sex, body mass index, alcohol consumption, education level, occupation, neighborhood socioeconomic status z-score, type of medical insurance, and smoking status. (DOCX 13 kb)
Additional file 2:
**Table A2.** Results from linear regression of distance to A1 or A2 roads, categorized as continuous (untransformed), inverse, and log-transformed distance truncated at 400 m, on markers of cardiac structure in JHS (*N* = 4826)^a^. ^a^Models adjusted for age, sex, body mass index, alcohol consumption, education level, occupation, neighborhood socioeconomic status z-score, type of medical insurance, and smoking status. (DOCX 12 kb)
Additional file 3:
**Table A3.** Results from linear or Logistic regression of residential distance to A1 or A2 road on markers of cardiac structure among participants in the Jackson Heart Study, excluding those with CVD (*N* = 2976).^a^. ^a^Models adjusted for age, sex, body mass index, alcohol consumption, education level, occupation, neighborhood socioeconomic status z-score, type of medical insurance, and smoking status. (DOCX 13 kb)
Additional file 4:
**Figure A1.** Association between LV mass index and natural log of residential distance to A1 or A2 roads among participants in the Jackson Heart Study, fitted using a natural spline with 3° of freedom for distance to A1 or A2, adjusting for covariates. Shaded area represents 95% confidence interval. (*N* = 4826).^a^. ^a^Adjusted for age, sex, body mass index, alcohol consumption, education level, occupation, neighborhood socioeconomic status z-score, type of medical insurance, and smoking status. (TIFF 1392 kb)
Additional file 5:
**Figure A2.** Association between LV end-diastolic diameter and natural log of residential distance to A1 or A2 roads among participants in the Jackson Heart Study, fitted using a natural spline with 3° of freedom for distance to A1 or A2, adjusting for covariates. Shaded area represents 95% confidence interval. (*N* = 4826).^a^. ^a^Adjusted for age, sex, body mass index, alcohol consumption, education level, occupation, neighborhood socioeconomic status z-score, type of medical insurance, and smoking status. (TIFF 1392 kb)
Additional file 6:
**Figure A3.** Association between LV end-systolic diameter and natural log of residential distance to A1 or A2 roads among participants in the Jackson Heart Study, fitted using a natural spline with 3° of freedom for distance to A1 or A2, adjusting for covariates. Shaded area represents 95% confidence interval. (*N* = 4826).^a^. ^a^Adjusted for age, sex, body mass index, alcohol consumption, education level, occupation, neighborhood socioeconomic status z-score, type of medical insurance, and smoking status. (TIFF 1392 kb)

## Abbreviations

BMI: Body mass index
CVD: Cardiovascular disease
DTR: Distance to roads
HF: Heart failure
JHS: Jackson Heart Study
LV: Left ventricular
LVMI: Left ventricular mass index
MESA: Multi-Ethnic Study of Atherosclerosis
MSA: Metropolitan statistical area
NO_2_: Nitrogen dioxide
NSES: Neighborhood socioeconomic status
PM_10_: Coarse particulate matter
PM_2.5_: Fine particulate matter
