# Supplementary material for: Residential distance to major roadways and cardiac structure in African Americans: cross-sectional results from the Jackson Heart Study
Source: Environ Health. 2017 Mar 8;16:21. doi: 10.1186/s12940-017-0226-4 (PMC5341411; doi:10.1186/s12940-017-0226-4)
Supplement: Additional file 1: — Table A1. Results from linear or logistic regression of distance to A1 or A2 roads, in categories of <100 m, 100–199 m, 200–399 m, ≥ 400 m on markers of cardiac structure in JHS (N = 4826)a. aModels adjusted for age, sex, body mass index, alcohol consumption, education level, occupation, neighborhood socioeconomic status z-score, type of medical insurance, and smoking status. (DOCX 13 kb) [file 12940_2017_226_MOESM1_ESM.docx]

# Table A1. Results from linear or logistic regression of distance to A1 or A2 roads, in categories of <100 m, 100-199 m, 200-399 m, ≥ 400 m on markers of cardiac structure in JHS (N=4826)^a^

| **Distance to A1 or A2 road** | **<100m (n=58)** | **100-199m (n=84)** | **200-399m (n=307)** | **≥400m (n=4377)** |
| --- | --- | --- | --- | --- |
| LVMI, g/m^2.7^, beta (95% CI) | -0.7 (-3.6, 2.2) | 1.8 (-0.6, 4.2) | -0.5 (-1.8, 0.8) | REF |
| LV hypertrophy, OR (95% CI) | 1.30 (0.53, 3.18) | 1.67 (0.86, 3.21) | 0.94 (0.59, 1.49) | REF |
| LV end-diastolic diameter, mm, beta (95% CI) | 0.3 (-0.9, 1.4) | 0.8 (-0.2, 1.7) | 0.3 (-0.2, 0.8) | REF |
| LV end-systolic diameter, mm, beta (95% CI) | 0.9 (-0.4, 2.1) | 1.4 (0.4, 2.5)* | 0.2 (-0.3, 0.8) | REF |
| **Distance to A1 road** | **<100m (n=14)** | **100-199m (n=37)** | **200-399m (n=194)** | **≥400m (n=4581)** |
| LVMI, g/m^2.7^, beta (95% CI) | -2.2 (-8.3, 3.8) | 3.3 (-0.3, 7.0) | -0.5 (-2.1, 1.2) | REF |
| LV hypertrophy, OR (95% CI) | NA | 1.58 (0.62, 4.05) | 0.97 (0.53, 1.75) | REF |
| LV end-diastolic diameter, mm, beta (95% CI) | 0.9 (-1.4, 3.3) | 1.4 (-0.01, 2.8) | -0.09 (-0.7, 0.5) | REF |
| LV end-systolic diameter, mm, beta (95% CI) | 1.5 (-1.1, 4.1) | 1.9 (0.4, 3.5)* | -0.3 (-1.0, 0.4) | REF |

^a^Models adjusted for age, sex, body mass index, alcohol consumption, education level, occupation, neighborhood socioeconomic status z-score, type of medical insurance, and smoking status.

*p<0.05
