# Supplementary material for: Residential distance to major roadways and cardiac structure in African Americans: cross-sectional results from the Jackson Heart Study
Source: Environ Health. 2017 Mar 8;16:21. doi: 10.1186/s12940-017-0226-4 (PMC5341411; doi:10.1186/s12940-017-0226-4)
Supplement: Additional file 2: — Table A2. Results from linear regression of distance to A1 or A2 roads, categorized as continuous (untransformed), inverse, and log-transformed distance truncated at 400 m, on markers of cardiac structure in JHS (N = 4826)a. aModels adjusted for age, sex, body mass index, alcohol consumption, education level, occupation, neighborhood socioeconomic status z-score, type of medical insurance, and smoking status. (DOCX 12 kb) [file 12940_2017_226_MOESM2_ESM.docx]

# Table A2. Results from linear regression of distance to A1 or A2 roads, categorized as continuous (untransformed), inverse, and log-transformed distance truncated at 400 m, on markers of cardiac structure in JHS (N=4826)^a^

| **Distance to A1 or A2 road** | **Continuous** | **Inverse** | **Truncated at 400m** |
| --- | --- | --- | --- |
| LVMI, g/m^2.7^, beta (95% CI) | 0.0 (-0.0002, 0.0001) | -2.2 (-6.7, 2.4) | 0.06 (-0.6, 0.7) |
| LV hypertrophy, OR (95% CI) | 1.00 (1.00, 1.00) | 1.09 (0.24, 4.90) | 0.91 (0.75, 1.10) |
| LV end-diastolic diameter, mm, beta (95% CI) | 0.0 (-0.0001, 0.0) | -0.4 (-2.2, 1.4) | -0.04 (-0.3, 0.2) |
| LV end-systolic diameter, mm, beta (95% CI) | 0.0 (-0.0001, 0.0) | 0.3 (-1.7, 2.2) | -0.2 (-0.5, 0.1) |
| **Distance to A1 road** | **Continuous** | **Inverse** | **Truncated at 400m** |
| LVMI, g/m^2.7^, beta (95% CI) | -0.0001 (-0.0002, 0.0) | -3.9 (-12.9, 5.2) | -0.04 (-1.7, 1.6) |
| LV hypertrophy, OR (95% CI) | 1.00 (1.00, 1.00) | Undefined | 1.16 (0.64, 2.10) |
| LV end-diastolic diameter, mm, beta (95% CI) | 0.0 (-0.0001, 0.0) | -0.3 (-3.9, 3.2) | -0.3 (-0.9, 0.4) |
| LV end-systolic diameter, mm, beta (95% CI) | 0.0 (-0.0001, 0.0) | 0.7 (-3.1, 4.6) | -0.5 (-1.2, 0.2) |

^a^Models adjusted for age, sex, body mass index, alcohol consumption, education level, occupation, neighborhood socioeconomic status z-score, type of medical insurance, and smoking status.

*p<0.05
