# Supplementary material for: Residential distance to major roadways and cardiac structure in African Americans: cross-sectional results from the Jackson Heart Study
Source: Environ Health. 2017 Mar 8;16:21. doi: 10.1186/s12940-017-0226-4 (PMC5341411; doi:10.1186/s12940-017-0226-4)
Supplement: Additional file 3: — Table A3. Results from linear or Logistic regression of residential distance to A1 or A2 road on markers of cardiac structure among participants in the Jackson Heart Study, excluding those with CVD (N = 2976).a. aModels adjusted for age, sex, body mass index, alcohol consumption, education level, occupation, neighborhood socioeconomic status z-score, type of medical insurance, and smoking status. (DOCX 13 kb) [file 12940_2017_226_MOESM3_ESM.docx]

# Table A3. Results from linear or logistic regression of residential distance to A1 or A2 road on markers of cardiac structure among participants in the Jackson Heart Study, excluding those with CVD (N = 2976)^a^

|  | **<150m (n=65)** | **150-299m (n=91)** | **300-999m**  **(n=745)** | **≥1000m (n=2075)** | **Log-transformed distance to road (continuous)** |
| --- | --- | --- | --- | --- | --- |
| LVMI, g/m^2.7^, beta (95% CI) | -0.37  (-2.4, 1.7) | -0.08  (-1.9, 1.7) | -0.7  (-1.4, 0.02) | REF | 0.2  (-0.08, 0.5) |
| LV hypertrophy, OR (95% CI) | 0.76  (0.31, 1.88) | 0.73  (0.30, 1.64) | 0.78  (0.54, 1.13) | REF | 1.04  (0.91, 1.20) |
| LV end-diastolic diameter, mm, beta (95% CI) | 0.9  (-0.1, 1.9) | -0.1  (-1.0, 0.7) | 0.002  (-0.3, 0.3) | REF | -0.005  (-0.1, 0.1) |
| LV end-systolic diameter, mm, beta (95% CI) | 1.2  (0.2, 2.3)* | -0.3  (-1.2, 0.6) | -0.07  (-0.4, 0.3) | REF | -0.02  (-0.2, 0.1) |

^a^Models adjusted for age, sex, body mass index, alcohol consumption, education level, occupation, neighborhood socioeconomic status z-score, type of medical insurance, and smoking status.

*p<0.05
