# Supplementary figures and images for: Residential distance to major roadways and cardiac structure in African Americans: cross-sectional results from the Jackson Heart Study
Source: Environ Health. 2017 Mar 8;16:21. doi: 10.1186/s12940-017-0226-4 (PMC5341411; doi:10.1186/s12940-017-0226-4)

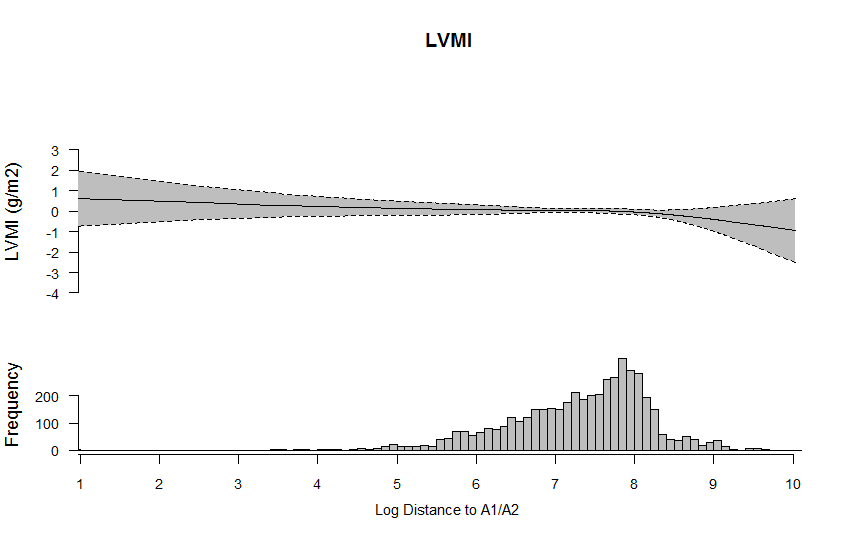

Supplement: Additional file 4: — Figure A1. Association between LV mass index and natural log of residential distance to A1 or A2 roads among participants in the Jackson Heart Study, fitted using a natural spline with 3° of freedom for distance to A1 or A2, adjusting for covariates. Shaded area represents 95% confidence interval. (N = 4826).a. aAdjusted for age, sex, body mass index, alcohol consumption, education level, occupation, neighborhood socioeconomic status z-score, type of medical insurance, and smoking status. (TIFF 1392 kb) [file 12940_2017_226_MOESM4_ESM.tiff]

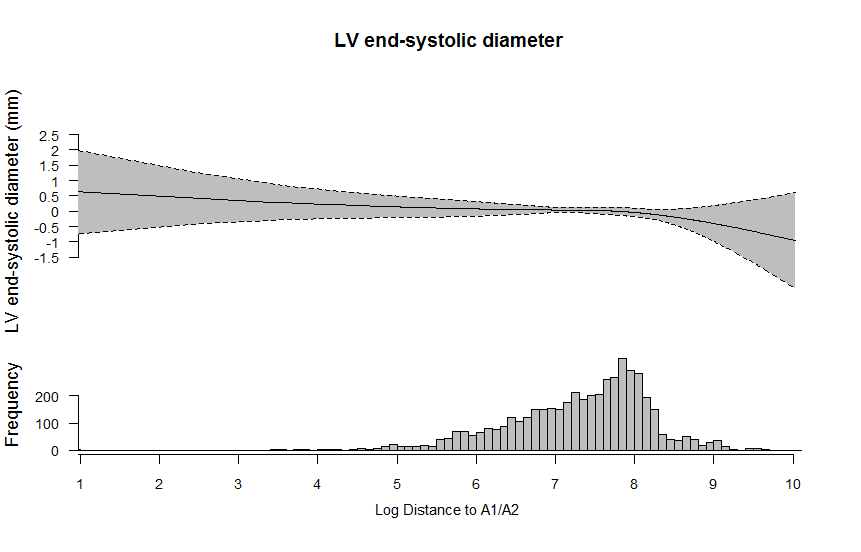

Supplement: Additional file 5: — Figure A2. Association between LV end-diastolic diameter and natural log of residential distance to A1 or A2 roads among participants in the Jackson Heart Study, fitted using a natural spline with 3° of freedom for distance to A1 or A2, adjusting for covariates. Shaded area represents 95% confidence interval. (N = 4826).a. aAdjusted for age, sex, body mass index, alcohol consumption, education level, occupation, neighborhood socioeconomic status z-score, type of medical insurance, and smoking status. (TIFF 1392 kb) [file 12940_2017_226_MOESM5_ESM.tiff]

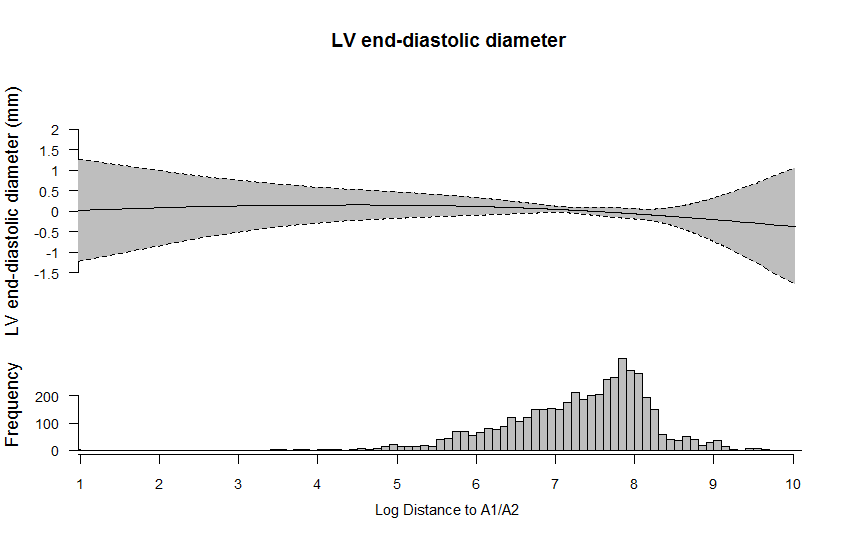

Supplement: Additional file 6: — Figure A3. Association between LV end-systolic diameter and natural log of residential distance to A1 or A2 roads among participants in the Jackson Heart Study, fitted using a natural spline with 3° of freedom for distance to A1 or A2, adjusting for covariates. Shaded area represents 95% confidence interval. (N = 4826).a. aAdjusted for age, sex, body mass index, alcohol consumption, education level, occupation, neighborhood socioeconomic status z-score, type of medical insurance, and smoking status. (TIFF 1392 kb) [file 12940_2017_226_MOESM6_ESM.tiff]
